# Host Suitability for Crapemyrtle Bark Scale (*Acanthococcus lagerstroemiae*) Differed Significantly among Crapemyrtle Species

**DOI:** 10.3390/insects12010006

**Published:** 2020-12-23

**Authors:** Bin Wu, Runshi Xie, Gary W. Knox, Hongmin Qin, Mengmeng Gu

**Affiliations:** 1 Department of Horticultural Sciences, Texas A&M University, College Station, TX 77843, USA; bin.wu@tamu.edu (B.W.); fushe001@tamu.edu (R.X.); 2 Department of Environmental Horticulture, University of Florida/IFAS North Florida Research and Education Center, Quincy, FL 32351, USA; gwknox@ufl.edu; 3 Department of Biology, Texas A&M University, College Station, TX 77843, USA; 4 Department of Horticultural Sciences, Texas A&M AgriLife Extension Service, College Station, TX 77843, USA

**Keywords:** crapemyrtle bark scale, host range, host suitability, susceptibility, future breeding programs, *Lagerstroemia* spp., *Lythrum californicum*

## Abstract

**Simple Summary:**

An exotic insect, crapemyrtle bark scale (CMBS, *Acanthococcus lagerstroemiae*), has spread across 14 states of the U.S. The infestation of CMBS has negatively impacted the growth, flowering, and even fruiting of some Lythraceae plants to various extent, including cultivars of *Lagerstroemia* *indica*, *L. fauriei*, and *Punica granatum*. This raises concerns that CMBS would threaten other crapemyrtle species and native Lythraceae plants. Understanding the host range and the host suitability for CMBS would help evaluate the potential risks to landscapes and other ecosystems. Information on the host suitability provides beneficial information for breeding resistant cultivars. In this study, we conducted a host range test on six *Lagerstroemia* species (*L. caudata*, *L. fauriei* ‘Kiowa’, *L. indica* ‘Dynamite’, *L. limii*, *L. speciosa*, and *L. subcostata*) and a native Lythraceae plant in California (California loosestrife, *Lythrum californicum*) over 25 weeks. The infestation of CMBS was observed on all the tested Lythraceae plants. The suitability for CMBS differed significantly among the *Lagerstroemia* species. *Lagerstroemia limii* was the most suitable, whereas *L. speciosa* was the least suitable. This study expands the current knowledge on the host range for CMBS. Our results suggest that *L. speciosa* could be utilized in developing new cultivars with low CMBS suitability.

**Abstract:**

Crapemyrtle bark scale (CMBS, *Acanthococcus lagerstroemiae*), an invasive polyphagous sap-sucking hemipteran, has spread across 14 states of the United States since 2004. The infestation of CMBS has negatively impacted the flowering of ornamental plants and even the fruiting of some crops. Host identification is critical for determining potential risks in ecosystems and industries and helps develop strategic management. A host confirmation test was performed over 25 weeks using six *Lagerstroemia* species (*L. caudata*, *L. fauriei* ‘Kiowa’, *L. indica* ‘Dynamite’, *L. limii*, *L. speciosa*, and *L. subcostata*) and California loosestrife (*Lythrum californicum*). The 25-week observations confirmed all tested plants as the hosts. The repeated measures of analysis of variance (ANOVA; Tukey’s HSD, α = 0.05) indicated that the average number of CMBS females differed significantly between *L. limii* and *L. speciosa*. The highest number of the females observed on *L. limii* was 576 ± 25 (mean ± SE) at 17 weeks after inoculation (WAI), while the highest number was 57 ± 15 on *L. speciosa* at 19 WAI. In addition, *L. subcostata* and *L. speciosa* had significantly high and low numbers of males, respectively, among the *Lagerstroemia* species. Our results suggest that *L. speciosa* could be incorporated in developing new cultivars with low CMBS suitability.

## 1. Introduction

Plant germplasm evaluations are helpful for breeding cultivars that are resistant to diseases and insects. For disease resistance, powdery mildew (*Erysiphe lagerstroemia*) resistant *Lagerstroemia fauriei* was incorporated in crapemyrtle breeding programs, and many interspecific hybrids (*L. indica* × *fauriei*) were released with powdery mildew resistance [1,2,3,4]. Many crapemyrtle species, hybrids, and cultivars were evaluated for host suitability or potential resistance to crapemyrtle aphid (CMA, *Sarucallis kahawaluokalani*) [5,6], flea beetle (*Altica litigata*) [7], or Japanese beetle (*Popillia japonica*) [8]. Subsequently, the pure *Lagerstroemia* indica cultivar ‘Carolina Beauty’ was found to be less CMA-preferred than *L. indica* × *L. fauriei* hybrids [5], but more susceptible to flea beetles [7] and Japanese beetles [8] than interspecific cultivars with *L. fauriei*. Based on this information, breeders and growers can more effectively select or target CMA-resistant or beetle-resistant cultivars.

Crapemyrtle bark scale (CMBS, *Acanthococcus lagerstroemiae*), a sap-feeding insect mainly found on crapemyrtle plants, is originally from Asia and has also been reported in the United Kingdom [9,10,11,12,13,14,15,16,17]. Unfortunately, this exotic insect pest spread to the United States, probably due to the increasing volume and speed of foreign trade [18,19]. The infestation of CMBS has already occurred in 14 states, including Alabama, Arkansas, Georgia, Kansas, Louisiana, Mississippi, New Mexico, North Carolina, Oklahoma, South Carolina, Tennessee, Texas, Virginia [20], and Washington (personal communication). Identifying suitable plant hosts of this exotic insect is critical for determining potential risks to other species’ ecosystem stability [21,22]. Further, confirming the insect’s host range can help develop strategic management practices [23].

**Figure 1:**
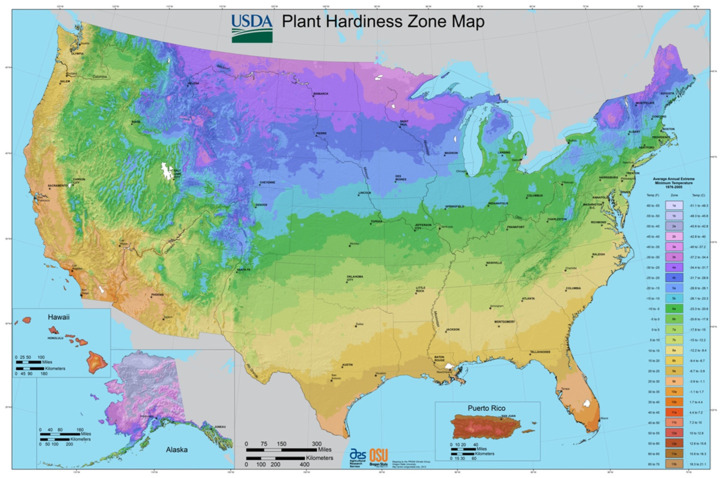
U.S. Department of Agriculture Plant Hardiness Zone Map. It is “the standard by which gardeners and growers can determine which plants are most likely to thrive at a location. This map is based on the average annual minimum winter temperature, divided into 10 °F zones” [32].

When it was first reported in Richardson, TX in 2004, the CMBS was tentatively recognized as azalea bark scale, *Eriococcus azalea* Comstock [24,25]. It was then identified as *Acanthococcus* (=*Eriococcus*) *lagerstroemiae* (*Kuwana*) based on both genetic and morphological evidence [26]. The CMBS is a highly fecund hemimetabolous insect [17]. The average number of eggs that a CMBS female lays ranges from 114 to 320 [27]. Similarly to other scale insects [28,29], newly hatched nymphs or crawlers develop as alate males through five nymphal stages, or as wingless females after three nymphal stages [17]. The males are covered by a white tubular sac during the prepupal and pupal stage, and the females are covered by a white oval-shaped ovisac after being fertilized [17,27]. The yearly number of generations varies from two to four depending on the geographic location and the climate [17,30,31]. The United States Department of Agriculture (USDA) Hardiness Zone shows the average annual minimum winter temperature relevant to plant growth and survival (Figure 1) [32]. In China, two generations of CMBS occurred per year from 1980 to 1983 in Shandong Province (USDA Hardiness Cold Zone 7) [16]. In Korea, two or three generations were observed per year in Jeonnam Province or Gyeongbuk Province (Zone 8) [10,13]. Temperature is a crucial factor affecting the adaptation and diversification of insects. To understand the potential distribution range of CMBS, its thermal tolerance was evaluated using higher and lower thermal limits, and it was predicted that CMBS could be limited by cold temperatures along the 43° N [33]. The physiology of CMBS was found to be associated with seasonally altered cold tolerance [34].

Because honeydew secreted from the ingested sap leads to the growth of black sooty mold covering the leaf surface and bark [31,35], CMBS threatens the growth and development of crapemyrtle and causes a reduction in aesthetic quality, resulting in concerns for landscapers and nursery growers [36]. Commercial insecticides are sometimes utilized to minimize CMBS infestations [37,38]. However, since they flower from late spring to early fall when few other resources are available [39,40], crapemyrtles are a good pollen source for pollinators, and are vital in ecosystem services benefiting humankind [41,42,43,44]. Consequently, insecticide applications on crapemyrtles to control this pest could severely affect the pollinators [45,46,47]. Non-chemical management of CMBS, such as resistance breeding and utilizing natural enemies, would be beneficial.

Natural infestations have been reported on not only crapemyrtles, but also on a wide range of plants from different families. A host plant is defined as a plant on which an insect is observed to complete its life cycle, especially with the presence of ovipositing gravid females [48,49]. Crapemyrtle bark scale exploits *Punica granatum* as a host, which seriously impacts the growth and fruiting of pomegranate and even leads to plant death [16,27,50,51]. It was also reported to feed on *Buxus microphylla* var. *koreana*, *Celtis sinensis*, *Diospyros kaki*, *Ficus carica*, *Hypericum kalmianum*, *Ligustrum obtusifolium*, *Mallotus japonicus*, *Malus pumila*, *Myrtus* sp., and *Prunus serrulata* and *Rubus* sp. [9,10,13,52,53]. In our previous study, infestations of CMBS were further confirmed on *Malus angustifolia*, *Malus domestica*, *Chaenomeles speciosa*, *Diospyros rhombifolia*, *Heimia salicifolia*, *Lagerstroemia* ‘Spiced Plum’, and twelve pomegranate cultivars [54]. Thus, CMBS is considered as a polyphagous insect with a relatively wide host range. In addition to *L. indica*, *L. fauriei*, and the interspecific hybrids, other crapemyrtle species, such as *L. limii*, *L. subcostata*, *L. caudata*, and *L. speciosa*, have been introduced into the United States as ornamental plants. To better manage CMBS in the U.S. and to help estimate its risks to ecosystems or green industries (wholesale and retail nurseries and landscape firms), further confirmation of CMBS hosts is necessary.

Currently, no CMBS-resistant crapemyrtle species or cultivars have been reported. Based on our previous observations [55], it is reasonable to predict that no *L. indica*, *L. fauriei*, or interspecific cultivars are immune to CMBS infestation. Infestation by CMBS was observed on nine crapemyrtle cultivars (Acoma, Basham’s Party Pink, Catawba, Country Red, Muskogee, Natchez, Sarah’s Favorite White, Sioux, and Tuscarora) in both landscapes and controlled environments. In addition, CMBS was observed on ten crapemyrtle cultivars (Biloxi, Burgundy Cotton, Chocataw, Lipan, Miami, New Orleans, Pink Ruffles, Powhatan, Royalty, and Tuskegee) in landscapes. *Lythrum alatum*, a plant in the same family (Lythraceae) as crapemyrtles, was reported as a CMBS host [17,30]. California loosestrife (*Lythrum californicum*) is native to California and is also distributed in Arizona, Kansas, New Mexico, Nevada, Oklahoma, Texas, and Utah. If *Ly. californicum* is indeed a host plant, its wide distribution will probably provide a continuum for spreading of CMBS. However, the suitability of *Ly. californicum* for CMBS is not yet known.

The aims of this study were to confirm additional plant hosts for CMBS and to test the host suitability among six *Lagerstroemia* species (*L. caudata*, *L. fauriei* ‘Kiowa’, *L. indica* ‘Dynamite’, *L. limii*, *L. speciosa*, and *L. subcostata*). The identification of less suitable species provides important information for breeding new CMBS-resistant cultivars.

## 2. Materials and Methods

### 2.1. Test Plants

Six *Lagerstroemia* species (*L. caudata*, *L. fauriei* ‘Kiowa’, *L. indica* ‘Dynamite’, *L. limii*, *L. speciosa*, *L. subcostata*) and *Ly. californicum* were tested in the study (Table 1). *Lagerstroemia caudata* and *L. indica* ‘Dynamite’ plants were donated by Dr. Cecil Pounders (Innovative Plants, LLC, Decatur, AL, USA) and Blake Jones in Georgia. *Lagerstroemia fauriei* ‘Kiowa’ plants were purchased from The Crape Myrtle Company (Archer, FL, USA). *Lagerstroemia limii, L. speciosa*, and *L. subcostata* were propagated from plants at North Florida Research and Education Center (Quincy, FL, USA). All plants (ranging from 50.8 to 88.9 cm in height) were transplanted into 3.79 L pots containing Jolly Gardener Pro-Line C/25 growing mixture (Oldcastle Lawn and Garden Inc, Poland Spring, ME, USA) and put inside plant cages (75 cm × 50 cm × 40 cm) in March 2019 before CMBS inoculation. The cage was made of PVC pipe, covered, and enclosed with handmade Chiffon mesh netting (Fabric Wholesale Direct, Farmingdale, NY, USA), and a 30-cm-long zipper was added to water and observe plants easily.

### 2.2. CMBS and Host Range Test

**Figure 2:**
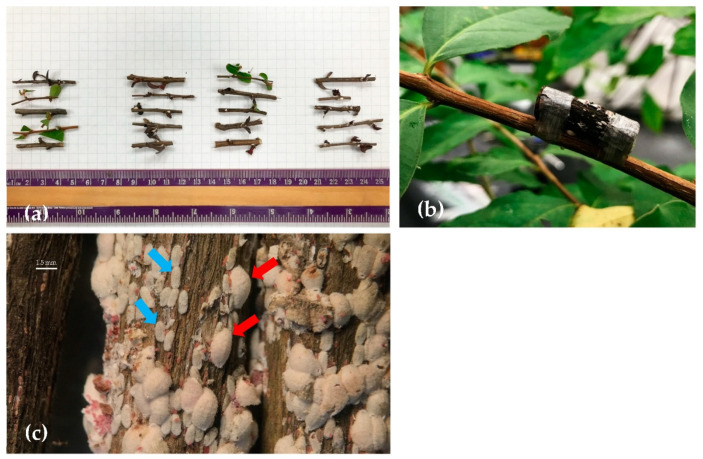
Crapemyrtle bark scale inoculation on six *Lagerstroemia* species and *Lythrum californicum* in one cage. (**a**) Three-centimeter-long CMBS-infected branches were collected from the nursery pad at the Department of Horticultural Sciences in Texas A&M University. (**b**) A CMBS-infected branch was tied on *L. subcostata*. (**c**) A closer look at the CMBS-infected branches; CMBS males (blue arrows) were recognized by white tubular sacs, and females (red arrows) were recognized by white round spindle-shaped ovisacs.

The experiment was conducted in the Department of Horticultural Sciences greenhouse at Texas A&M University (30°36′31.9″ N, 96°21′1.9″ W. A set of the seven species mentioned above was enclosed in one cage and inoculated with CMBS-infested branches. The cage was replicated three times. Crapemyrtle branches infested with CMBS (Figure 2a) were collected from the nursery at the Department of Horticultural Sciences of Texas A&M University in May 2019. Before the branches were attached to each test plant using Parafilm^®^, all except five ovisacs on the branches were removed (Figure 2b). To ensure successful CMBS inoculation, each test plant was tied with newly collected branches containing five fresh ovisacs again five weeks after the initial inoculation. Cages were placed on different benches, approximately 2.5 m in distance, in the greenhouse at 25 ± 5 °C and 50 ± 10% relative humidity under a 10.5 h L: 13.5 h D photoperiod. The CMBS males were recognized by snow-white tubular sacs (Figure 2c) and females were recognized by white round spindle-shaped ovisacs [17]. The numbers of the males and females, respectively, per plant were observed weekly for the first three weeks and then counted biweekly from three weeks after the first-time inoculation (WAI) until 25 WAI.

### 2.3. Statistical Analysis

The experiment was arranged in a randomized complete block design with plant species being one treatment factor. Each of the experimental units was measured biweekly for 25 weeks, so the data collection time was the second treatment factor. Each cage was a block, and there were three blocks.

Log transformation as log_10_((No. of CMBS) + 1) was conducted prior to data analysis. The numbers of males and females on different species over 25 weeks were analyzed as repeated measures, respectively, using analysis of variance (ANOVA) with a mixed effect in JMP Pro 15 (SAS Institute, Cary, NC, USA). Plant species and data collection time were assigned with full factorial. The blocks were included as a random effect. Then, the least squares means (LSMeans) of the number of the CMBS on species were separated using Tukey’s honestly significant difference (HSD) (α = 0.05). When needed, original data prior to log transformation or reverse-transformed data were presented. Graphs were plotted using GraphPad Prism 8 (GraphPad Software, San Diego, CA, USA).

## 3. Results

### 3.1. Host Range Confirmation

**Figure 3:**
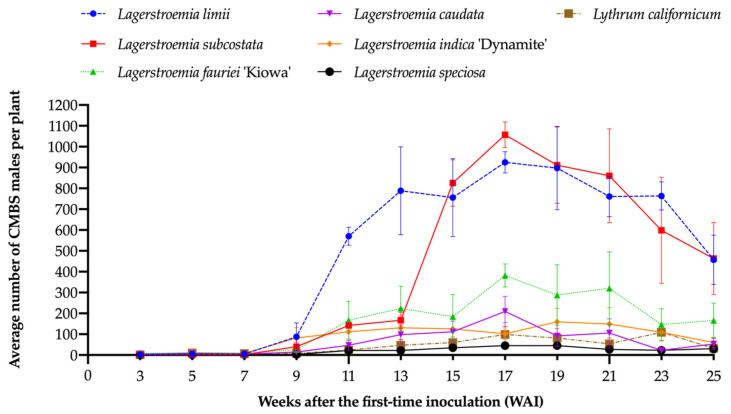
Changes of the average number of CMBS males that emerged on the six *Lagerstroemia* species and *Ly. californicum* over 25 weeks. Bars are standard errors of the mean.

**Figure 4:**
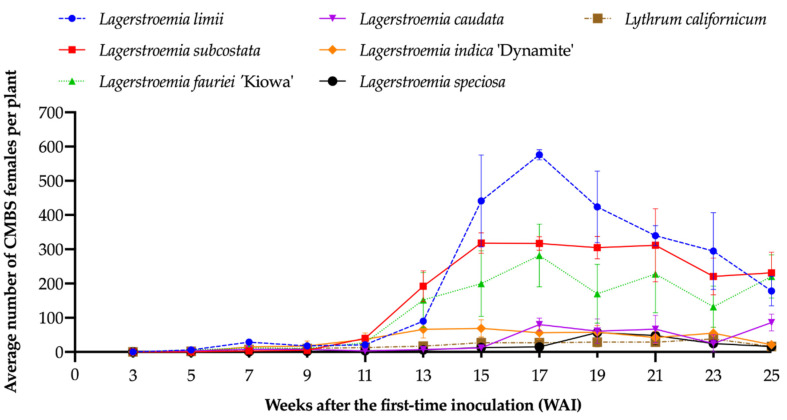
Changes of the average number (Mean ± SE) of CMBS females that emerged on six *Lagerstroemia* species and *Ly. californicum* over 25 weeks.

**Figure 5:**
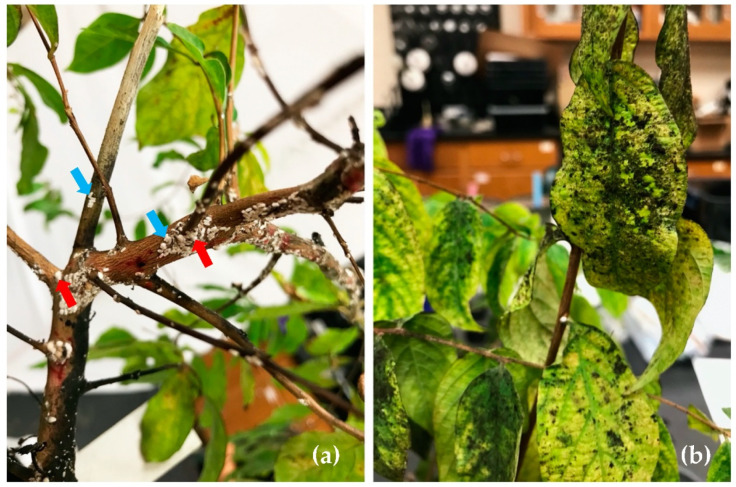
The infestation of CMBS occurred on *Lagerstroemia subcostata* and *Lagerstroemia limii* after inoculation. (**a**) Numerous CMBS males (blue arrows) and females (red arrows) developed on stems of *L. subcostata*, and black sooty mold accumulated on the bark 13 weeks after CMBS inoculation. (**b**) Black sooty mold accumulated on leaves of *L. limii*.

The CMBS males were first observed on *L. fauriei* ‘Kiowa’ at two WAI. Beginning at three WAI (29 May 2019), white sacs were first observed on *Ly. californicum* and all other *Lagerstroemia* species. Meanwhile, the females were first seen on *Ly. californicum* and all *Lagerstroemia* species except *L. speciosa* and *L. subcostata* at five WAI, and were observed on all species at seven WAI. Average numbers of CMBS males and females increased and peaked around 17 WAI on most species. The number of the males decreased at 19 WAI (Figure 3) and female densities decreased at 21 WAI (Figure 4). Because the life cycle of CMBS is around six weeks [17,30], CMBS on all test plants would have completed at least one life cycle (the period roughly goes: eggs → nymphs → adult females (fertilized by males) → laying eggs), which confirmed that all test plant species were accepted by CMBS, and that they were CMBS hosts. Black sooty mold resulting from honeydew excreted by CMBS was observed on the bark or leaves of all *Lagerstroemia* plants at 17 WAI (Figure 5). No black sooty mold was observed on CMBS-infected *Ly. californicum*.

### 3.2. The Suitability for CMBS Differed Significantly among the Lagerstroemia Species

The number of CMBS reflects the host suitability for CMBS among *Lagerstroemia* species. There was no interaction between species and time affecting the number of CMBS males (*F* = 1.42; df = 55,132; *p* = 0.0558). The fixed-effect test showed that the main factors, plant species (*F* = 3.96; df = 5,12; *p* = 0.0236) and time (*F* = 50.1; df = 11,132; *p* < 0.0001), had significant effects on the number of CMBS males. Based on the 25-week comparison results using Tukey’s HSD (Table 2), the LSMeans of the average number of the males on *L. speciosa* was significantly lower than on the other six species (*L. limii* and *L. subcostata, L. fauriei* ‘Kiowa’ and *L. indica* ‘Dynamite’, and *L. caudata*). However, the LSMeans among these other species had no significant difference over the 25 weeks. According to the average number of CMBS (Figure 3), the highest number of the males on *L. subcostata* was 1057 ± 107 (mean ± SE) at 17 WAI, whereas the highest number on *L. speciosa* was 45 ± 29 (mean ± SE) at 19 WAI.

The species–time interaction (*F* = 1.62; df = 55,132; *p* = 0.0135), time (*F* = 71.78; df = 11,132; *p* < 0.0001), and plant species (*F* = 4.28; df = 5,12; *p* = 0.0182) had significant effects on the number of CMBS females. The simple-effect differences among the species at each measuring time were examined. At 3, 5, 7, 9, or 11 weeks after inoculation (WAI), no difference was observed in the number of female CMBS among different species. At 13, 15, 17, 19, 21, 23, and 25 WAI, the number of female CMBS from different species was significantly different (Figure 4).

The LSMeans of the number of the females on *L. limii* (63) was significantly higher than that on *L. subcostata* (49), *L. fauriei* ‘Kiowa’ (30), and *L. indica* ‘Dynamite’ (16), followed by *L. caudata* (7) as well as *L. speciosa* (7) (Table 2). The highest average number of females on *L. limii* was 576 ± 25 (mean ± SE) at 17 WAI, and the number on *L. speciosa* peaked at 57 ± 15 (mean ± SE) at 19 WAI (Figure 4). These *Lagerstroemia* species showed significantly different host suitability for *A. lagerstroemiae*.

During the 25-week experiment, the sex ratio of CMBS on *L. subcostata*, *L. indica* ‘Dynamite’, *L. limii*, *L. fauriei* ‘Kiowa’, *L. caudata*, and *L. speciosa* was 2.8:1, 2.5:1, 1.9:1, 1.6:1, 2.7:1, and 1.6:1, respectively (Table 2). No evidence showed the association of the number of CMBS with the sex ratio among these plant species.

### 3.3. The Effect of the Species–Time Interaction on the Weeks after Inoculation (WAI) when the Number of CMBS Increased Significantly Compared to the Previous Week on Different Species

The number of males on *L. limii*, *L. subcostata*, or *L. indica* ‘Dynamite’ did not increase significantly compared to the previous week until 11 WAI (*L. fauriei* ‘Kiowa’ at 13 WAI) (Table 3 and Table S1). There was no significant increase in the number of the males on *L. caudata* or *L. speciosa* between consecutive weeks during the 25-week experiment. The number of CMBS females on *L. limii*, *L. subcostata*, *L. fauriei* ‘Kiowa’, or *L. indica* ‘Dynamite’ did not become significantly higher compared to the previous week until 11 WAI (Table 3 and Table S1), and then, there was no significant change in insect densities among these crapemyrtle species. The number on *L. caudata* or *L. speciosa* did not increase significantly compared to the previous week until 17 WAI, representing six weeks later than the more suitable crapemyrtle species.

## 4. Discussion

The hosts confirmed in this study validated *Lagerstroemia indica*, which agrees with the CMBS hosts listed in Kozar’s findings [53], and *L. fauriei* (mentioned as *L. japonica* in the host list [15,53]). Moreover, this study added four additional *Lagerstroemia* species (*L. limii*, *L. caudata*, *L. speciosa*, and *L. subcostata*) and *Ly. californicum* as CMBS hosts.

One important finding is that *L. speciosa* is not suitable for the growth and development of CMBS. Among all tested crapemyrtle species, *L. speciosa* supported CMBS’s growth and development the least, as indicated by the lowest numbers of male and female CMBS (Table 2). The highest number of males on *L. speciosa* was nearly 23-fold less than that on *L. subcostata*. The largest peak of the females on *L. speciosa* was 10-fold less than that on *L. limii*. A previous feeding preference study found that *L. speciosa* was the least preferred host for crapemyrtle aphids [5]. Thus, it is reasonable to predict that *L. speciosa* is not suitable for the growth and development of phloem-sap hemipterans.

An interesting observation from this study was the different sex ratio of CMBS on different crapemyrtle species. Even though the number of male CMBS on *L. speciosa* did not differ significantly over the 25 weeks (Table 3), the number of females (fertilized by male CMBS) on *L. speciosa* increased significantly from 15 to 17 WAI, which was six weeks later than on the more suitable species (*L. subcostata*, *L. fauriei* ‘Kiowa’, and *L. indica* ‘Dynamite’). The sex ratio (male–female) of CMBS on *L. speciosa* (1.6:1) was much lower than on other tested species, such as *L. subcostata* (2.8:1), which may restrict the occurrence of severe CMBS infestation on *L. speciosa* for a period. Herbivore sex ratios can be affected by environmental factors, host plant defensive chemistry, and nutrient availability [66,67,68,69,70]. Our results showed different sex ratios of CMBS on different crapemyrtle species, which hinted at the importance of the male insect’s contribution to individual reproduction or the population dynamics via the quality of nuptial gifts [71,72].

Different levels of host suitability could be attributed to, but not limited to, physical properties [6], a balance between stimulation and deterrence [73,74], and some secondary metabolites of the plant [75,76]. For example, many alkaloids, terpenoids, flavonoids, sterols, and polyphenols in different structural types have been isolated from various parts of different *Lagerstroemia* species, such as *L. indica*, *L. subcostata*, *L. fauriei*, and *L. speciosa* [77,78,79]. Currently, there is no report on the association between CMBS suitability and plant compounds. To further understand the CMBS–host interaction, one important future direction would be to investigate the role of plant compounds on host suitability and biological parameters of *A. lagerstroemiae*, which would help improve the integrated pest management for CMBS.

## 5. Conclusions

This study confirmed *L. limii*, *L. caudata*, *L. speciosa*, *L. subcostata*, and *Ly. californicum* as CMBS hosts in addition to the previously reported *L. indica* ‘Dynamite’ and *L. fauriei*. Importantly, these *Lagerstroemia* species showed significantly different suitability to CMBS. *Lagerstroemia speciosa* was the least suitable for CMBS, as indicated by the lowest numbers of CMBS males and females, and can be utilized as a parental plant for breeding new CMBS-resistant cultivars.

## Figures and Tables

**Table 1:** Seven plant species evaluated as host candidates of crapemyrtle bark scale (CMBS).

Plant Species	Recommended U.S. Department of Agriculture Plant Hardiness Zone	Native Origin	Mature Height (m) *
*Lagerstroemia caudata*	9–10	China (Guangdong, Guangxi, Jiangxi)	18.0–30.0 [56]
*L. fauriei* ‘Kiowa’	6–9	Japan	3.0–4.6 [57,58]
*L. indica* ‘Dynamite’	6–10	The USA (Oklahoma)	4.6–6.0 [59,60]
*L. limii*	8–10	China (Fujian, Hubei, and Zhejiang)	4.0–7.0 [61]
*L. speciosa*	9–13	China (Fujian, Guangdong, Guangxi, Hainan, Yunnan), India, Malaysia, Philippines, Sri Lanka, Vietnam	20.0–40.0 [62,63]
*L. subcostata*	4–11	Japan (Ryukyu Islands), China (Anhui, Guangdong, Guangxi, Hubei, Hunan, Jiangxi, Jiangsu, Qinghai Sichuan, Taiwan, Zhejiang)	6.1 to 9.1 [64]
*Lythrum californicum*	4–9	Northern Mexico, central USA (Arizona, California, Kansas, New Mexico, Nevada, Oklahoma, Texas)	Up to 1.5 [65]

* Numbers inside brackets are references.

**Table 2:** The least squares means of the male and female *Acanthococcus lagerstroemiae* and sex ratio (male–female) on different *Lagerstroemia* species within 25 weeks after inoculation.

Plant Species	No. Males (95% CI ^z^)	No. Females (95% CI)	Sex Ratio
*L. subcostata*	139 (47–405) a ^y^	49 (18–131) ab	2.8:1
*L. limii*	119 (40–348) ab	63 (24–167) a	1.9:1
*L. fauriei* ‘Kiowa’	48 (16–140) ab	30 (11–80) ab	1.6:1
*L. indica* ‘Dynamite’	40 (13–118) ab	16 (6–43) ab	2.5:1
*L. caudata*	19 (6–56) ab	7 (2–20) ab	2.7:1
*L. speciosa*	11 (3–33) b	7 (2–19) b	1.6:1

^z^ CI = confidence intervals. ^y^ Log transformation as log_10_((No. of CMBS) +1) was conducted prior to data analysis. The original number of CMBS and the reverse-transformed 95% CI values are presented. The numbers within a single column indicated by the same letter are not significantly different within 25 weeks, as compared by Tukey’s honestly significant difference (HSD) (α = 0.05).

**Table 3:** The weeks after inoculation (WAI) when the number of *Acanthococcus lagerstroemiae* males or females increased significantly compared to the previous week on different *Lagerstroemia* species.

Plant Species	The WAI When CMBS Significantly Increased	
	CMBS Males	CMBS Females
*Lagerstroemia subcostata*	11	11
*Lagerstroemia limii*	11	11
*Lagerstroemia fauriei* ‘Kiowa’	13	11
*Lagerstroemia indica* ‘Dynamite’	11	11
*Lagerstroemia caudata*	NA	17
*Lagerstroemia speciosa*	NA	17

Note: The WAI when the number of males and females significantly increased compared to the previous week on each species was distinguished using Tukey’s HSD (α = 0.05) during the 25-week experiment. ‘NA’ indicates that the average number of CMBS males did not change significantly during the experiment.

## Data Availability

The data presented in this study are available in the supplementary material attached in this article.

## Supplementary Materials

The following are available online at https://www.mdpi.com/2075-4450/12/1/6/s1, Table S1: The effect of the species-time interaction on the weeks after inoculation (WAI) when the number of CMBS males increased significantly compared to the previous week on different species, Table S2: The effect of the species-time interaction on the weeks after inoculation (WAI) when the number of CMBS females increased significantly compared to the previous week on different species.
